# In Vitro Cell Culture Infectivity Assay for Human Noroviruses

**DOI:** 10.3201/eid1303.060549

**Published:** 2007-03

**Authors:** Timothy M. Straub, Kerstin Höner zu Bentrup, Patricia Orosz Coghlan, Alice Dohnalkova, Brooke K. Mayer, Rachel A. Bartholomew, Catherine O. Valdez, Cynthia J. Bruckner-Lea, Charles P. Gerba, Morteza A. Abbaszadegan, Cheryl A. Nickerson

**Affiliations:** * Pacific Northwest National Laboratory, Richland, Washington, USA; † Tulane University School of Medicine, New Orleans, Louisiana, USA; ‡ University of Arizona, Tucson, Arizona, USA; § Arizona State University, Tempe, Arizona, USA; 1 Current affiliation: Arizona State University, Tempe, Arizona, USA

**Keywords:** norovirus, cytopathic effect, fluorescence, electron microscopy, 3-dimensional cell culture, organoids, RWV bioreactor, research

## Abstract

Human noroviruses cause severe, self-limiting gastroenteritis that typically lasts 24–48 hours. Because of the lack of suitable tissue culture or animal models, the true nature of norovirus pathogenesis remains unknown. We show that noroviruses can infect and replicate in a 3-dimensional (3-D), organoid model of human small intestinal epithelium. Cells grown on porous collage-coated beads under fluid shear conditions in rotating wall vessel bioreactors differentiate into 3-D architectures resembling both the morphologic and physiologic function of in vivo tissues. Microscopy, PCR, and fluorescent in situ hybridization provided evidence of norovirus infection. Cytopathic effect and norovirus RNA were detected at each of the 5 cell passages for genogroup I and II viruses. Our results demonstrate that the highly differentiated 3-D cell culture model can support the natural growth of human noroviruses, whereas previous attempts that used differentiated monolayer cultures failed.

## Abstract 2

A 3-dimensional organoid human small intestinal epithelium model was used.

Human noroviruses are the leading cause of nonbacterial, self-limiting gastrointestinal illness worldwide (*1*–*4*). Infected persons may develop symptoms of severe nausea, vomiting, and watery diarrhea within 12–24 hours of exposure and typically remain symptomatic for 1–2 days (*5*). Infections may lead to death for immunocompromised persons. The most common routes of norovirus transmission are ingestion of contaminated food and water and person-to-person contact (*5*).

Noroviruses are nonenveloped, positive-sense, single-stranded RNA viruses ≈27–35 nm in diameter (*6*,*7*). They belong to the genus *Norovirus* in the family *Caliciviridae* and consist of 3 genogroups (I, II, and IV) that infect humans (*8*–*11*). On the basis of sequence diversity of the capsid gene, noroviruses can be classified into 8 genetic clusters within GI, 17 in GII, and 1 in GIV (*11*).

Understanding of the pathogenesis of human noroviruses has been limited by our inability to propagate these viruses in vitro (*12*). Studies of viral attachment to cultured gastrointestinal epithelial cells (Caco-2) using recombinant virus-like particles or infectious noroviruses indicate that specific histo–blood group antigens play a key role in the attachment of the virus to the host cells (*13*–*17*).

Recently, the first in vitro norovirus cell culture model was reported for a virus that infects mice (*18*,*19*). Asanaka et al. (*20*) reported production of Norwalk virus particles (norovirus GI.1, the prototype strain of this genera from Norwalk, Ohio) after transinfection of cultured kidney cells. However, these models do not answer the fundamental questions of human norovirus attachment and entry into cells of the human gastrointestinal tract. In vitro differentiation of small intestinal epithelium that approaches physiologic functionality of the in vivo host may allow for the development of a pathogenesis model for norovirus.

Representative models of differentiated human intestinal epithelium can be established by growing cells in 3 dimensions (3-D) on collagen-I–coated porous microcarrier beads in rotating-wall vessel (RWV) bioreactors that model the physiologic fluid-shear environment in their respective organs (*21*–*24*). The design of the RWV bioreactor is based on the principle that organs and tissues function in a 3-D environment and that this spatial context is necessary for development of cultures that more realistically act like in vivo tissues and organs (*25*). We present the results of our first attempts to infect a physiologically relevant 3-D small intestinal epithelium model (INT-407) with genogroup I and II human noroviruses.

## Materials and Methods

### Generation of the Small Intestinal Epithelium Model

We summarize results from 4 different infectivity trials that used 3-D small intestinal epithelial cells (Table 1). The human embryonic intestinal epithelial cell line INT-407 was obtained from the American Type Culture Collection (Manassas, VA, USA) (CCL-6). It was initially grown as standard monolayers in GTSF-2 medium (Hyclone, Logan, UT, USA) containing penicillin/streptomycin and Fungizone (Invitrogen, Carlsbad, CA, USA) in T-75 flasks at 37°C in a 5% CO_2_ environment in preparation for seeding into the RWV. GTSF-2 medium is a triple-sugar minimal essential medium α-L-15 base supplemented with 10% fetal bovine serum, 2.2 g/L NaHCO_3_, and 2.5 mg/L insulin-transferrin-sodium selenite (*26*).

**Table 1:** Summary of methods from the 4 norovius infectivity trials*

Infectivity trial (date)	Virus stocks/comments†	Time points assayed	Assays performed
First (Mar 2005)	Combined equal volumes of strains 149, 155, and flag2 (P0). Effective dilutions of 10^–1^ to 10^–3^ were assayed. Supernates were harvested from all infected wells, dilutions of viral stock, and time points combined (≈15 mL final volume).	1 h–72 h; media changed in all wells at 24 h postinfection.	CPE, RT-PCR (Table 3), thin section light microscopy and ultrathin section TEM (Figure 1).
Second (Jun 2005)	Supernate cocktail from first infectivity trial (P1), strains 149 and flag2 tested alone (P0 stool samples). Supernates from each time point were harvested for subsequent infectivity trials (≈3 mL/time point).	Same as first infectivity trial; media changed every 24 h.	Same as first infectivity trial, except that CPE (Figure 2) was documented photographically (Figure 3 refers to TEM).
Third (Aug 2005)	Supernate from combined stock (P2), 149, and flag2 (P1), stool sample flag2 (P0) were harvested. Controls generated by ultrafiltration (10,000 MWCO).	Same as second infectivity trial.	Same as second infectivity trial (Figure 4); first attempt with FISH
Fourth (Dec 2005)	Infectivity followed through 5 passages in cell culture using strains 155 and flag2 (P0–P5). Effective dilution of viruses at P5 = 1:10^6^, if replication was not occurring.	Infected aggregates were processed at 24 h postinfection, and the viruses were used for subsequent passage.	CPE, RT-PCR, molecular beacon FISH (Figure 5). PCR products from P3 of both strains were cloned and sequenced. Compared sequences with sequenced PCRs from original stools.

*CPE, cytopathic effect; RT-PCR, reverse transcription–PCR; TEM, transmission electron microscopy; MWCO, molecular weight cutoff; FISH, fluorescence in situ hybridization.  †Passage no. (P#) definitions: P0, viruses from a stool sample and used to infect a cell culture for the first time; P1, viruses harvested from P0 cell cultures and used to infect cell culture a second time; P2, viruses harvested from P1 and used to infect cell culture a third time; P3, viruses harvested from P2 and used to infect cell culture a fourth time; P4, viruses harvested from P3 and used to infect cell culture a fifth time; and P5, viruses harvested from P4 and used to infect cell culture a sixth time.

Cells were trypsinized at 70% confluency, and 5 × 10^6^ cells were added to the RWV. Cells were assayed for viability by trypan blue dye exclusion. Then they were introduced into the RWV (Synthecon, Inc, Houston, TX, USA) containing 5 mg/mL porous Cytodex-3 microcarrier beads (collagen type-I–coated porous microspheres, average size 175 microns in diameter, Sigma, St. Louis, MO, USA), which produced a final ratio of 10 cells/bead (*21*,*22*). Cells were cultured in the RWV bioreactors, with the rotation speed adjusted to maintain the cell aggregates in suspension during the entire culture duration (≈18–20 rotations/min initial and 24–28 rotations/min final, depending on the size of the aggregates).

Cell growth was monitored daily by measurement of pH, dissolved CO_2_ and O_2_, and glucose use by using a Corning blood gas analyzer (Model 168; Corning, NY, USA) and a Beckman Glucose Analyzer-2 {Beckman-Coulter, Fullerton, CA, USA). Fresh medium was replenished by 90% of the total vessel volume each 24–72 hours, depending on the growth and metabolic rate of the cultures. 3-D cells were harvested 35 days after seeding into the RWV except for the fourth infectivity trial, for which aggregates were harvested starting on day 29 and continuing to day 35. Using a 10-mL wide-bore pipette, mature 3-D aggregates were placed into 24-well plates (40,000 cells/well) and infected with norovirus on the same day they were harvested.

Before each infectivity assay, immunohistochemical staining was performed on aliquots of the 3-D INT-407 cells to ensure differentiation. Aliquots of the mature tissue aggregates were fixed with paraformaldehyde (4% paraformaldehyde in 1× phosphate-buffered saline [PBS]) for 30 min at room temperature and then stained with antibodies specific for tight junction markers ZO-1, Occludin, Claudin-1, and E-cadherin (Zymed Laboratories [Invitrogen], South San Francisco, CA, USA). The aliquots were then imaged using confocal laser scanning microscopy (Zeiss Axioplan II microscope, Carl Zeiss, Thornwood, NY, USA). Correct localization of these markers at cell membranes is highly indicative of differentiated cells (*22*). Previous characterization of the 3-D INT-407 model also included collagen type-II, fibronectin, sialyl Lewis A antigen, villin, and periodic acid–Schiff staining to show mucin production (*22*).

Viruses, diluted 1:5 to 1:1,000 in GTSF-2 media, were applied as 0.1-mL aliquots per well across a minimum of 3 wells per time point for each of the infection trials described in Table 1. Viruses were introduced to the cells by gentle mixing of the aggregates with the viral suspension. The infected aggregates were incubated for 1 h at 37^o^C in a 5% CO_2_ incubator before being overlaid with 1 mL of fresh GTSF-2 media.

### Preparation and Characterization of Virus Stocks

Stool samples were obtained from persons who became ill during acute gastroenteritis outbreaks on cruise ships (identified as samples 149 [GII] and 155 [GI]) and in a nursing home (identified as flag2 [GII]). Approximately 1 g of stool was suspended in 0.01 M PBS to obtain a 10%–20% stool suspension (≈5–10 mL). The suspension was vortexed for 60 s, centrifuged at 1,000× *g*, and processed through a 0.22-micron filter to remove bacterial contamination. Virus samples were stored at –80°C for future assays.

The presence of norovirus in the purified samples was confirmed by reverse transcription–PCR (RT-PCR) and sequenced (*10*). BLAST (www.ncbi.nlm.nih.gov/BLAST) was performed on these sequences to determine genogroup (Table 2). Stool extracts were screened for other enteric viruses by 3 passages on Buffalo Green Monkey cells and Caco-2 cells grown as conventional monolayers. Stool extracts were also tested for enterovirus by RT-PCR (*27*).

**Table 2:** Genetic characterization of the RNA-dependent RNA polymerase sequence of the norovirus strains used in the study

Sample ID	Date collected (2004)	Setting	Strain in GenBank with closest sequence similarity (%)	Genogroup
149	Apr 14	Cruise ship	AJ487474 NLV/Castell/2001/Sp (97%)	II
155	Jun 21	Cruise ship	DQ157140 Hu/Offenburg1155/2004/D (100%)	I
flag2	Dec 14	Nursing home	AJ626578 NV/GII/Stockholm/IV1138/2003/SE (97%)	II

### Microscopic Analysis

Cellular pathology of 3-D tissue aggregates for the second and third infection trials was documented by using an Olympus DP70 CCD camera and inverted microscope system (Nikon Eclipse TE300, Kanagawa, Japan) at each time point assayed. Subcellular pathology was assessed by using light and transmission electron microscopy (TEM). These samples (≈40,000 cells per well) were fixed in 3.5% glutaraldehyde/0.5% paraformaldehyde in PBS and processed by washing cells 3× with 0.1 M sodium cacodylate buffer (Electron Microscopy Sciences, Hatfield, PA, USA) before incubation in 1% osmium tetroxide diluted in 0.1 M sodium cacodylate buffer for 1 h at room temperature. Cells were washed with buffer and dehydrated by using a graded series of ethanol rinses (33, 50, 75, 90, and 3 × 100% ethanol). Samples were then embedded in hard-grade LR WhiteTM Resin (London Resin Co., Berkshire, England) at 60^o^C for 24 h. Block faces were cut into the samples by using a Leica EM Trim (Wetzlar, Germany). Thin (60 nm) and ultrathin (30 nm) sections were cut by using a Diatome Ultra 45º (Biel, Switzerland) diamond knife on a Leica Ultracut UCT ultramicrotome. Thin sections were affixed to microscope slides and stained with toludine blue, then viewed on a Nikon Optiphot-2 light microscope. Ultrathin sections were affixed to copper mesh grids, stained and contrasted with uranyl acetate and lead citrate for 7 min each, and then viewed on a JEOL-2010 (Tokyo, Japan) transmission electron microscope at 106 kV.

### Viral RNA Extraction and RT-PCR

RNA from tissue samples was extracted by using either an RNEasy or a ViralAmp RNA extraction kit (Qiagen, Valencia, CA, USA). RT-PCR was performed by using the OneStep RT-PCR kit (Qiagen) according to manufacturers’ instructions. Primer sequences for RT-PCR and seminested PCR to amplify the RNA-dependent RNA polymerase gene are listed in Vinje et al. (*10*), with the exception of the MP290 primer for seminested PCR, which is 5′-GAYTACTCYCSITGGGAYTC-3′. Viral RNA was subjected to RT-PCR for 60 min at 42º C and 15 min at 95º C to inactivate the RT enzyme and activate Taq. A 3-step PCR was then conducted for 40 cycles (30 s at 94ºC, 30 s at 50ºC, and 30 s at 72º C).

### Sequencing

PCR products amplified from cell cultures (P3 passage) of the fourth infection trial were sequenced after cloning into the PGEM-T Easy Vector System (Promega, Madison, WI, USA). Sequences have been deposited in GenBank under accession nos. DQ531707 (for outbreak sample 155) and DQ531708 (for outbreak sample flag2).

### Fluorescence in Situ Hybridization

The molecular beacon fluorescence in situ hybridization (FISH) assay used during the fourth infection trial used modified reverse PCR primer sequences for genogroup I and II viruses (*28*). For genogroup 1, the modified probe sequence was 5′-TAMRA-CAGGCCCTTAGACGCCATCATCATTGCCTG-DABCYL-3′, and for genogroup 2, the modified probe sequence was 5′-TAMRA-CTCGGTCGACGCCATCTTCATTCACACCGAG-DABCYL-3′. (Underlined sequences for each probe indicate the self-complementary regions.)

Cells in the tissue aggregate were fixed in 4% paraformaldehyde for 30 min and then washed 3 times in 1× Dulbecco’s PBS (DPBS, Sigma). The weight of the aggregates allowed these to settle by normal gravity to the bottom of the microfuge tube. Tissues were permeabilized with 0.1% Triton X-100 in 1× DPBS for 15 min at room temperature and then washed 3× with 1× DPBS. Molecular beacon (either genogroup 1 or genogroup 2) was suspended to a final concentration of 1 μM in 1× DPBS. The molecular beacon was incubated with the tissues in a water bath for 1 h at 37ºC. The aggregates were then washed 3× with 1× DPBS and transferred to 12-well tissue culture plates. Cell aggregates were imaged by using a Leica confocal laser scanning microscope with a 63× water immersion objective. Captured images were digitally stacked to create 3-D images (VOLOCITY, Improvision Inc., Lexington, MA, USA).

## Results

### First Infection Trial (March 2005)

**Figure 1:**
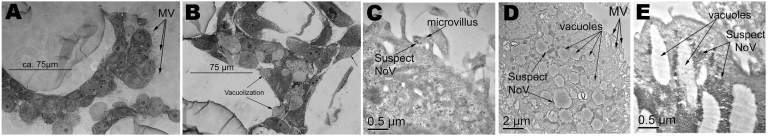
Light and transmission electron micrographs of uninfected and infected tissue aggregates with a combined stock of noroviruses representing 3 strains (Passage 0 [P0]). A) Uninfected tissue aggregates displaying well-formed microvilli. B) Infected tissue aggregates exhibiting vacuolization and shortening of the microvilli. C) Transmission electron microscopy (TEM) at 1 h postinfection showing possible norovirus in a microvillus. D) TEM at 24 h postinfection showing significant vacuolization, and internal membrane rearrangement. E) TEM at 66 h postinfection showing accumulation of suspect norovirus particles.

This first attempt was performed with a cocktail of norovirus strains 149, 155, and flag2 (stool samples are defined as Passage 0 [P0]). At 24 h postinfection, infected cell aggregates exhibited vacuolization and shortening of the microvilli (Figure 1) and were detached from the cytodex beads, exhibiting cytopathic effect (CPE) (data not shown). CPE was first observed in tissue aggregates receiving the highest concentration of virus and then developed in aggregates receiving the lowest concentration of virus. TEM showed the presence of uniform 29-nm–diameter particles, consistent with the size of norovirus particles, which invaded the microvilli within 1 h postinfection (Figure 1, Panel C) and accumulated within the tissue aggregates within 24–66 h postinfection (Figure 1, Panels D and E, respectively). Concomitant with microscopic observations, viral RNA was detected as early as 1 h postinfection for the 1:10 and 1:100 dilutions and at 66 h postinfection for the 1:1,000 dilution (Table 3).

**Table 3:** Relative increases in viral RNA as measured by limiting dilution PCR*

Effective dilution of working viral stock applied to cells	Hours postinfection			
	1	24	66	72
1:10	+	+	+	ND
1:100	+	+	+	ND
1:1,000	-	-	+	+
Negative control	-	-	-	-

*ND, not done.

### Second Infection Trial (June 2005)

**Figure 2:**
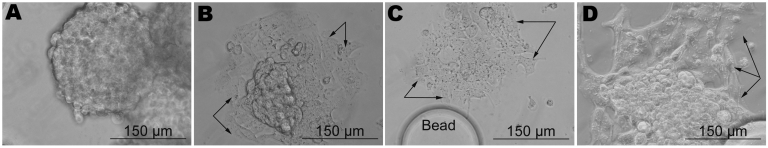
Demonstration of cytopathic effect in infected tissue aggregates during the second infection trial. A) Uninfected aggregate, 24 h into the experiment. B) Cells infected with lysate from the first infection trial (P1) at 24 h postinfection. C) Stool sample flag2 at 24 s postinfection (P0). D) Stool sample 149 at 48 h postinfection (P0). Arrows indicate cells exhibiting unusual pathology.

**Figure 3:**
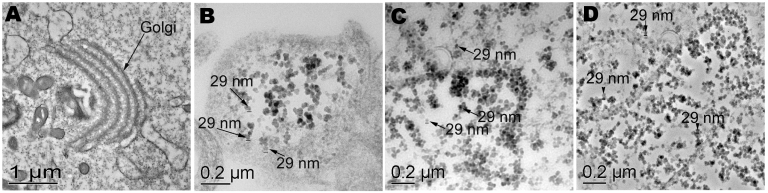
Transmission electron microscopy of uninfected and infected cell cultures from the second infection trial. A) Uninfected cells showing normal internal membrane organelles. B) Suspect 29-nm particles in cells, viruses from cell culture lysate from the first infection trial (P1). C) Stool sample flag2 (P0) and D) stool sample 149 (P0) showing numerous 29-nm particles and internal rearrangement of membrane-bound organelles.

Figures 2 and 3 show results for CPE and TEM, respectively, for P1 of the combined virus stocks and P0 for strains flag2 and 149 (Figure 2, Panels B, C, and D and Figure 3, Panels B, C, and D). As with the first infection trial, viral CPE was manifested by cells sloughing off the collagen beads as a mat, with the individual cells becoming highly elongated or distorted within 24–48 h postinfection. Similar to other studies that used murine norovirus (*18*), TEM exhibited uniformly sized 27–29-nm particles in infected cell aggregates and internal membrane rearrangement. With RT-PCR, norovirus RNA was detected in all infected samples. Both the combined stock (P1) and flag2 (P0) showed an increase of viral RNA as detected by limited dilution during RT-PCR.

### Third Infection Trial (August 2005)

**Figure 4:**
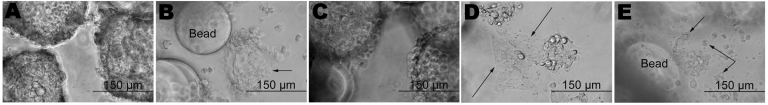
Cytopathic effect results from the third infectivity trial. A) Virus-free control of B) combined viral stock lysate from second passage experiment (second infectivity trial, P1), which was used to infect naive cells (P2). C) Virus-free control of the flag2 stool sample. D) Corresponding infection with the flag2 stool sample (P0). E) Flag2 in cell culture (P1). Cells in Panels B, D, and E were confirmed as positive for norovirus by reverse transcription–PCR (RT-PCR) and seminested PCR. Cells in uninfected controls were negative for norovirus by both RT-PCR and nested PCR. Arrows indicate cells exhibiting unusual pathology.

In trial 3, we used P2 of the virus cocktail (strains 149, 155, and flag2), P1 of flag2, and the stool sample from flag2 (P0) to infect a new batch of differentiated 3-D INT-407 cells. For virus-negative controls, we filtered the virus inoculum through a 10,000–molecular weight cutoff (MWCO) filter. Both the cocktail and the flag2 isolate were able to generate CPE within 24 h postinfection (Figure 4, Panels B, D, and E), whereas the MWCO filtrates did not show CPE and tested negative by RT-PCR (Figure 4, panels A and C).

### Fourth Infection Trial (December 2005)

**Figure 5:**
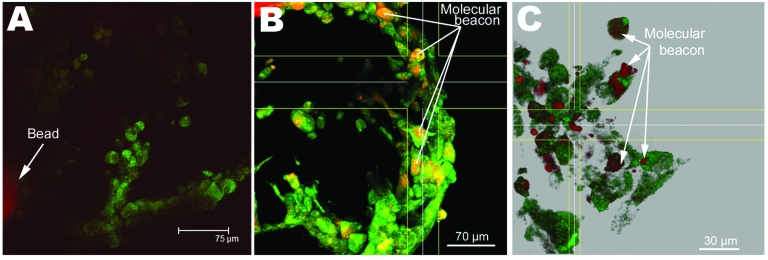
Deconvolved confocal laser scanning micrographs of the molecular beacon fluorescence in situ hybridization assay, demonstrating viral infectivity of a genogroup I virus (Sample 155) and genogroup II virus (flag2). A) Typical response for uninfected cells, no molecular beacon observed. B) Sample 155, P5 in cell culture. C) Sample flag2, P5 in cell culture.

In trial 4, we used strain 155 (genogroup I) and flag2 (genogroup II) and infected 3-D INT-407 cells. We followed viral infection for both of these strains through 5 passages in cell culture. With each viral passage, cell cultures showed CPE after 24–48 h, and norovirus RNA for both strains was detected by FISH with genogroup specific molecular beacons (Figure 5). We further sequenced RT-PCR products from the original stool sample that contained strains 155 and flag2 and both strains from passage 3 in cell culture. Only 1 nucleotide substitution for passage 3 flag2 was observed in a 261 bp product, and no nucleotide change was shown for strain 155.

## Discussion

Our primary goal was to develop an in vitro cell culture assay for human noroviruses. This assay is necessary before we can even begin to understand the mechanisms of pathogenesis. Our starting point for developing an infectivity assay for human noroviruses was to use the 3-D INT-407 small intestinal epithelium model previously developed for the study of *Salmonella* pathogenesis (*22*). Multiple factors were considered for choosing this model. First, early biopsy studies that used human volunteers indicated that norovirus infection targets the human small intestine (*29*,*30*). Second, reports showing differentiation of INT-407 cells in 3-D in the RWV essentially produces a “co-culture” model of multiple intestinal cell types (enterocytes, goblet cells, and M cell–like markers) (*22*). This phenomenon of multicellularity has been hypothesized as a factor likely needed for norovirus infectivity (*12*). Finally, extensive characterization of this model 3-D system (*22*,*23*) showed apical expression of certain cell-surface antigens (e.g., Lewis antigen A), which are thought to be important in the attachment of noroviruses to cells (*4*,*13*–*17*,*31*).

However, expression of these antigens only is not sufficient for a successful cell culture of human norovirus because attempts to infect 3-D aggregates from Caco-2 and HT-29 cells were unsuccessful (data not shown). We are not sure whether this phenomenon is due solely or in part to 1) correct presentation of the cell surface receptors that would be necessary for viral attachment and efficient entry into cells or 2) physiologic relevance of the 3-D small intestinal model that confirms previous human biopsy studies that show human noroviruses have an affinity for cells of the small intestine (*29*,*30*).

We have developed the first successful in vitro cell culture assay for norovirus based on multiple lines of orthogonal evidence. CPE has been 1 measure of viral infectivity, but this measure alone can be deceiving. Duizer et al. (*12*) noted CPE in several samples but on further investigation found that it was caused by contaminating viruses. For the 3 virus strains we investigated, we took several measures to ensure that the viruses were indeed noroviruses. First, patients from these 3 outbreaks showed clinical symptoms typical of norovirus infection. Second, these virus isolated failed to produce CPE through 3 passages in conventional monolayers of buffalo green monkey and Caco-2 cells. Third, RT-PCR for co-infecting enteroviruses was negative. Finally, successful norovirus replication was demonstrated through 5 passages in the 3-D small intestinal model, as determined by CPE, RT-PCR, and FISH.

Although the Duizer et al. (*12*) study noted infrequent CPE , likely due to contaminating viruses, our study demonstrated positive CPE every time viruses came in contact with cell culture and norovirus-positive RT-PCR, regardless whether they came from stool samples or at any passage number in the 3-D INT-407 cell culture. Furthermore, positive CPE from a viral sample could be extinguished by passing the sample through an ultrafilter. Additionally, light microscopy and TEM demonstrated both the pathology and evidence of accumulation of viral particles that are the correct size for human norovirus. We confirmed that these particles were human norovirus by RT-PCR, sequencing, and FISH with genogroup-specific molecular beacons.

In vitro cell culture models used to study the host-pathogen interaction have benefited from the recognition that organs and tissues function in a 3-D environment and that this spatial context is necessary for development of cultures that more realistically resemble the in vivo tissues and organs from which they were derived (*21*–*23*). We used RWV bioreactor technology to engineer 3-D models of human small intestinal epithelium to investigate susceptibility for norovirus infection. This method to generate 3-D organoid models has been used to study *Salmonella* *typhimurium* and *Escherichia coli* infection by using small and large intestinal models (*21*–*23*,*32*), *Pseudomonas* infection by using lung epithelial models (*33*), cytomegalovirus infection by using placental tissue models (*34*), and Epstein-Barr virus by using lymphoblastoid cell models (*35*).

Our study shows that selecting the appropriate cell line, growing the samples as 3-D aggregates, and infecting them when they are fully differentiated is key for successful in vitro cell culture of human noroviruses. Future research with this model will include further testing of a broader panel of genetically diverse human noroviruses, determining the sensitivity, identifying neutralizing epitopes and protective immune responses, and obtaining a better understanding of the molecular biology of norovirus replication and transcription to develop improved prevention protocols.
